# Protocol: a simple gel-free method for SNP genotyping using allele-specific primers in rice and other plant species

**DOI:** 10.1186/1746-4811-6-12

**Published:** 2010-04-21

**Authors:** Naoki Hirotsu, Naomi Murakami, Takayuki Kashiwagi, Kazuhiro Ujiie, Ken Ishimaru

**Affiliations:** 1 Division of Plant Sciences, National Institute of Agrobiological Sciences, Kannondai 2-1-2, Tsukuba, Ibaraki 305-8602, Japan; 2 Current address: Department of Life Sciences, Faculty of Life Sciences, Toyo University, 1-1-1 Izumino, Itakura, Gunma 374-0193, Japan; 3 Current address: Department of Bioproductive Science, Faculty of Agriculture, Utsunomiya University, 350 Mine, Utsunomiya, Tochigi 321-8505, Japan

## Abstract

**Background:**

Genotype analysis using multiple single nucleotide polymorphisms (SNPs) is a useful but labor-intensive or high-cost procedure in plant research. Here we describe an alternative genotyping method that is suited to multi-sample or multi-locus SNP genotyping and does not require electrophoresis or specialized equipment.

**Results:**

We have developed a simple method for multi-sample or multi-locus SNP genotyping using allele-specific primers (ASP). More specifically, we (1) improved the design of allele-specific primers, (2) established a method to detect PCR products optically without electrophoresis, and (3) standardized PCR conditions for parallel genomic assay using various allele-specific primers. As an illustration of multi-sample SNP genotyping using ASP, we mapped the locus for lodging resistance in a typhoon (*lrt5*). Additionally, we successfully tested multi-locus ASP-PCR analysis using 96 SNPs located throughout the genomes of rice (*Oryza sativa*) cultivars 'Koshihikari' and 'Kasalath', and demonstrated its applicability to other diverse cultivars/subspecies, including wild rice (*O. rufipogon*).

**Conclusion:**

Our ASP methodology allows characterization of SNPs genotypes without electrophoresis, expensive probes or specialized equipment, and is highly versatile due to the flexibility in the design of primers. The method could be established easily in any molecular biology laboratory, and is applicable to diverse organisms.

## Introduction

Plant biologists frequently have to analyze the genotype of multiple polymorphic loci. For multi-sample or multi-locus genotyping, many researchers employ labor-intensive methods, such as cleaved amplified polymorphic sequences (CAPS) [1], restriction fragment length polymorphisms (RFLP) [2], and simple sequence repeats (SSR) [3]. The main difficulty in multiple genotyping using such genetic markers is that it requires much time and labor, since these analyses need multi-step sample processing including electrophoresis. Moreover, there are only few of the appropriate polymorphisms in some genome regions [4]. To fill in gaps between markers, single nucleotide polymorphisms (SNPs) are available for genotyping.

Advances in genome analysis have made it possible to utilize SNPs in *Arabidopsis thaliana *[5], *Glycine max *[6], and *Zea mays *[7]. In rice (*Oryza sativa*), the genome sequence of the ssp. *japonica *cultivar 'Nipponbare' [8] and the draft sequence of the ssp. *indica *cultivar '93-11' [9] have been determined, and genome-wide SNP maps of *japonica *and *indica *have been published [4,10-12]. Yu et al. [9] identified, on average, one SNP per 170 bp throughout the genome in rice. These highly abundant SNPs will greatly facilitate high-resolution genome-wide genotyping [13]. Based on information provided by Nasu et al. [10] and Monna et al. [12], a database containing SNPs of six *japonica *and two *indica *cultivars was released (Rice SNPs Database http://www.pgcdna.co.jp/snps/index.html) by the Plant Genome Center Co. Ltd. (Tsukuba, Japan).

The allele-specific primer PCR (ASP-PCR) method was developed for allele analysis of clinically significant mutations [14]. Allele-specific PCR primers, designed so that their 3' terminal nucleotides correspond to an SNP, match perfectly with one allele (the specific allele) but have a 3' mismatch with other alleles (the nonspecific alleles). ASPs preferentially trigger amplification of the specific allele [15], and the presence of the SNP can be detected as PCR amplification after electrophoresis. Although each of the ASP-PCR markers is a dominant marker, pairs of ASP for both alleles can be used as codominant markers. To facilitate highly reliable discrimination between two alleles, the addition of artificial mismatches at the third base from 3'end of the primers might be beneficial [15-17]. Their reliability was demonstrated in *Arabidopsis thaliana *[18] and wheat [19]. However, these methods require electrophoresis for detection, and the insufficient information of the mismatch base for new primer design is available. Thus, the application of ASP-PCR has been restricted so far. To meet the needs of multi-sample or multi-locus SNP genotyping, the ASP-PCR method clearly requires improvement.

In this study, we introduce such improvements of the ASP-PCR method. First, to discriminate SNP alleles through the presence or absence of amplification, high amplification specificity was enforced. Second, for multi-sample SNP genotyping, a detection method not requiring electrophoresis was developed. Third, for genotyping multi-locus SNPs in a single PCR operation, we standardized allele-specific PCR conditions among ASP-PCR markers. Finally, we demonstrated the usefulness of the ASP-PCR methodology for multi-sample or multi-locus SNP genotyping.

## Materials

### Plant materials and SNP information

The SNPs between two rice cultivars 'Koshihikari' (*japonica*) and 'Kasalath' (*indica*) were used in this study. The SNPs information of 'Koshihikari' and 'Kasalath' were provided by the PGC SNPs Database System http://www.pgcdna.co.jp/snps/. The marker names with 'S' are SNPs in intergenic region [10,12] and 'T' are SNPs in coding region [20]. The physical map loci of SNP markers were determined using NCBI blast http://www.ncbi.nlm.nih.gov/BLAST/. Genomic DNA was isolated from leaf blades by the cetyl trimethyl ammonium bromide (CTAB) method [21].

### Equipment

Standard laboratory equipment including a thermal cycler and an UV transilluminator is required for allele-specific primer PCR. A real-time thermal cycler is optionally desirable for quantitative analysis of SYBR green I fluorescence. All our PCR were performed on iCycler thermal cycling instrument (Bio-rad, Hercules, CA), and SYBR Green I fluorescence was detected using a UV transilluminator (TFML-30E, UVP, CA) or a TP800 Thermal Cycler Dice (Takara Bio Inc., Shiga, Japan).

## Protocols

### Primer design

SNP assays are performed by pairs of PCR amplifications, one with 'Koshihikari' allele specific primer and the other with 'Kasalath' allele specific primer; the reverse primer is non-allele specific and identical in 'Koshihikari' and 'Kasalath' allele specific PCRs. The allele-specific forward primer should be designed so that their 3' terminal nucleotides correspond to an SNP. To improve the specificity of allele-specific amplification, single nucleotide artificial mismatches (A-G transition as well as A-T, A-C, and G-T transversions) should be introduced at the third nucleotide from the 3' end of the primers.

### Demonstration of ASP-PCR

**Figure 1:**
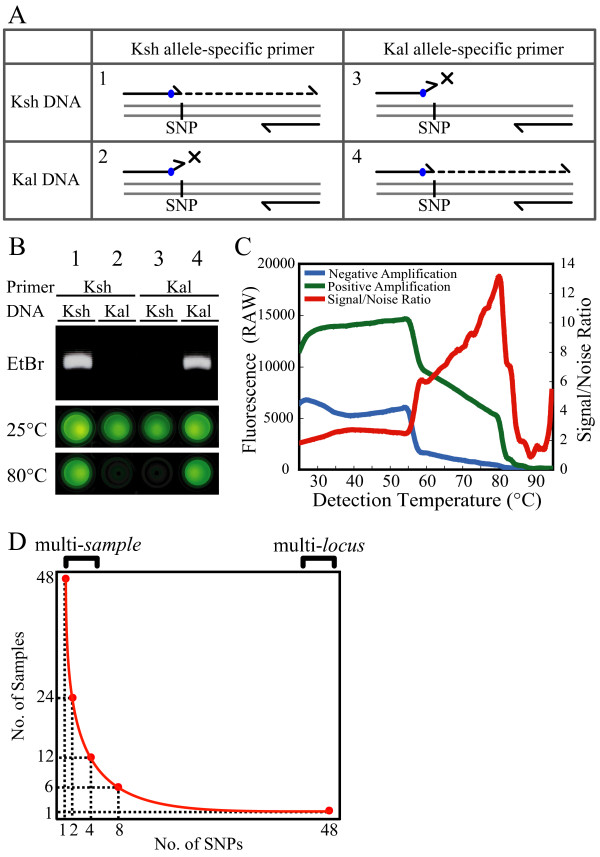
**Allele-specific amplification and detection of the PCR products**. (**A**) Schematic representation of the allele-specific primer PCR method. 'Koshihikari' (Ksh) allele specific primer forms a perfect match at the 3' end (SNP) with Ksh DNA sequence (1) but forms a mismatch with Kal DNA (2). 'Kasalath' (Kal) allele specific primer similarly forms a 3' end match with Kal DNA (4) and 3' end mismatch with Ksh DNA (3). Both allele specific primer has an artificial mismatch at third base from 3' end (blue circle) according to the result from Table 1. (**B**) Allele-specific amplification of SNP marker S0285 detected EtBr after gel electrophoresis. Fluorescence of same samples were detected with a UV transilluminator at room temperature (25°C) or immediately after heating to 80°C using SYBR Green I. Ksh allele-specific primers (lane 1 and 2) and Kal allele-specific primers (lane 3 and 4) were used for PCR of Ksh genomic DNA (lane 1 and 3) and Kal genomic DNA (lane 2 and 4). The specificity of the reaction is evident. (**C**) The effect of temperature on SYBR Green I fluorescence. The green line indicates the fluorescence intensity of the sample in which amplification had occurred, and the blue line shows the amplification-independent background fluorescence. The ratio of both (signal/noise ratio) is shown in red. (**D**) The relationship between the number of SNPs and samples in a single PCR operation using 96-well plate. In a single PCR operation, 48 samples SNP genotype could be examined (multi-sample, Fig. 2), or 48 locus SNPs of one sample could be examined (multi-locus, Fig. 3)

Fig. 1A, B shows an example of allele-specific amplification for SNP marker S0285 (chr. 5, 44.7 cM). 'Koshihikari' allele-specific primers (lane 1 and 2) and 'Kasalath' allele-specific primers (lane 3 and 4) were used for allele-specific PCR with 'Koshihikari' genomic DNA (lane 1 and 3) and 'Kasalath' genomic DNA (lane 2 and 4). Amplification was detected using ethidium bromide (EtBr) after electrophoresis. 'Koshihikari' allele-specific primers acted only on 'Koshihikari' genomic DNA, and 'Kasalath' allele-specific primers worked only in the presence of 'Kasalath' genomic DNA.

### Optical detection of allele-specific amplification

To abolish the need for electrophoresis as an analytical step, SYBR Green I, which intercalates into double-stranded DNA [22], was added to the solution containing the PCR products. Ethidium bromide has been used for the detection of double-stranded DNA, but this compound exhibits high intrinsic fluorescence and does not allow for specific double-stranded DNA analysis [23]. SYBR Green I has low intrinsic fluorescence and high selectivity for double-stranded DNA [22], and thus helps to avoid a purification step to remove the non-bound dye. The intensity of fluorescence was monitored at various temperatures using a real-time thermal cycler (Fig. 1C). Fluorescence intensity was strongly temperature-dependent. Differential melting behavior of non-specific agglomerates and the amplification product lead to a significantly increased signal/noise ratio (> 6) in the range between 60°C and 80°C, ensuring reliable detection of the specific PCR product. Thus, allele-specific amplification was unambiguously detected by SYBR Green I using both UV transilluminator and real-time thermal cycler (Fig. 1B, C).

### Common protocol for ASP-PCR

1. Add the following components to a nuclease-free microcentrifuge tube:

12.3 μl nuclease-free water

2 μl 10 × PCR buffer

1.6 μl 2.5 mM dNTP mix

0.5 μl forward primer (10 μM)

0.5 μl reverse primer (10 μM)

1 μl sample DNA (ca. 200 ng).

0.1 μl Hot Goldstar DNA polymerase (catalogue No. ME-0073, Eurogentec, Seraing, Belgium)

2. Place reactions in a thermal cycler heat block and incubate 10 min denaturation at 95°C; 30 cycles of 30 s denaturation at 95°C, 30 s annealing at 58°C, 45 s extension at 72°C; and 4 min final extension at 72°C.

3. Add 2 μl 10 × SYBR Green I (catalogue No. 50513, Lonza, Basel, Switzerland) to the PCR product, and detect the fluorescence at 75°C.

**NOTE: ***The DNA polymerase and buffer system strongly influence the allele-specificity. The primers used in this study were optimized for Hot Goldstar DNA polymerase. We confirmed that some primers could be used in other polymerase (eg. SYBR Premix Ex Taq, catalogue No. RR041A, Takara, Tokyo, Japan)*.

### Application of ASP-PCR

In 96-well PCR plate, SNP genotyping in 48 samples can be examined in a single PCR operation (multi-sample). On the other hand, the number of SNPs can be increased by reducing the number of samples (multi-locus). We applied ASP-PCR to multi-sample SNP genotyping by mapping quantitative trait locus (QTL) for lodging resistance in a typhoon (*lrt5*) [24] region (Application 1) and multi-locus SNP genotyping by generating genome-wide graphical maps for 11 rice lines (Application 2).

### Application 1 (Multi-sample SNP genotyping)

As an example of multi-sample SNP genotyping, we mapped *lrt5 *region previously mapped within 28.6 cM region on chromosome 5 using a cross between 'Koshihikari' and 'Kasalath' [24]. We used 96 F_2 _segregating plants from crosses between *japonica *'Koshihikari' and 'S1', a NIL line containing 'Kasalath' chromosome segment at the *lrt5 *region. For mapping *lrt5*, we developed five ASP-PCR markers in *lrt5 *region (See additional file 1: Allele-specific PCR primers used to map the *lrt5*). These markers have different annealing temperatures individually.

#### Sub-protocol for multi-sample SNP genotyping

1. Prepare a PCR master mix by scaling the volumes listed below to the desired number of amplification reactions. Include 10% overage to cover pipetting errors.

• Add the following components to a nuclease-free microcentrifuge tube:

12.3 μl nuclease-free water

2 μl 10 × PCR buffer

1.6 μl 2.5 mM dNTP mix

0.5 μl forward primer (10 μM)

0.5 μl reverse primer (10 μM)

0.1 μl Hot Goldstar DNA polymerase

• Mix gently and centrifuge to bring solution to the bottom of the tube.

2. Aliquot 19 μl of the PCR master mix into 96-well PCR plate and add 1 μl sample DNA (ca. 200 ng).

3. Place reactions in a thermal cycler heat block and incubate 10 min denaturation at 95°C; 30 cycles of 30 s denaturation at 95°C, 30 s annealing at X°C, 45 s extension at 72°C; and 4 min final extension at 72°C.

**NOTE: ***The annealing temperature is changed to the temperature described in *additional file 1.

4. Add 2 μl 10 × SYBR Green I to the PCR product, and acquire the fluorescence value using a real-time thermal cycler at 75°C.

5. Determine the genotypes using Microsoft Excel macros which are programmed to identify the genotype according to the threshold of fluorescence intensity (to obtain these macro files, please contact to kenshi@nias.affrc.go.jp).

**Figure 2:**
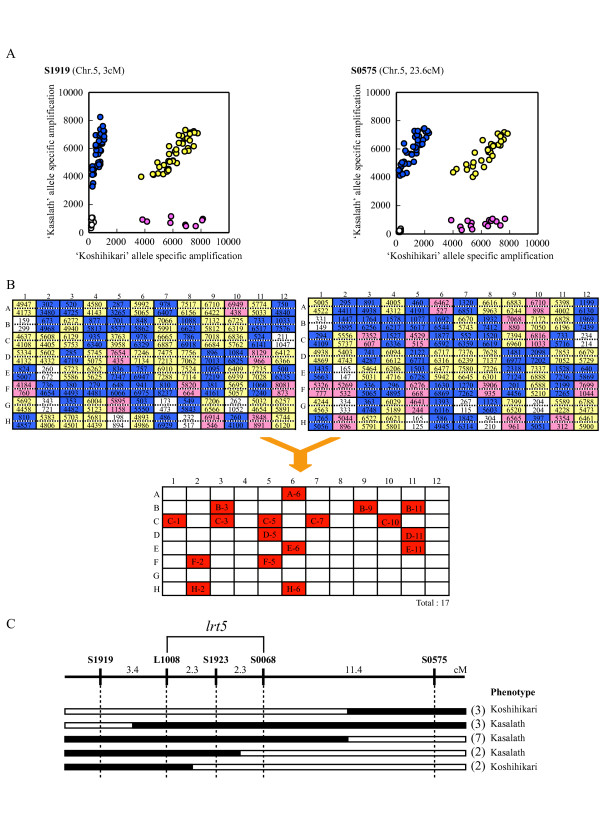
**An example of multi-sample SNP genotyping**. To demonstrate multi-sample SNP genotyping, we mapped the *lrt5 *region using an F_2 _population. (**A**) Scatter plots showing genotyping results for the S1919 (left) and S0575 (right) markers. The X- and Y-axis represent fluorescence intensity value of 'Koshihikari' and 'Kasalath' allele-specific amplification, respectively. (**B**) The fluorescence data of 'Koshihikari' (upper tier in each field) and 'Kasalath' (lower tier) that correspond to the data in (**A**) are shown in the upper two panels in the 96-well format. Red, blue and yellow indicate 'Koshihikari' homozygotes, 'Kasalath' homozygotes, and heterozygotes of the allele, respectively. In the lower panel, recombination events between the markers S1919 and S0575 are indicated in red. Recombination was determined from the genotypes indicated in the upper two panels. (**C**) Genetic map of the *lrt5 *region, derived from the genotypes of 17 recombinants as analyzed using three additional ASP-PCR markers. White and black bars represents 'Koshihikari' and 'Kasalath' alleles, respectively. Number of recombinants are given in parenthesis.

According to sub-protocol for multi-sample SNP genotyping, we first analyzed S1919 and S1975 markers. Eight plants did not show PCR amplification due to germination error. The genotype of each SNP was determined by the presence or absence of fluorescence; homozygotes of the 'Koshihkari' and 'Kasalath' alleles and heterozygotes were clearly discriminated (Fig. 2A). Seventeen plants that showed recombination events could be detected (Fig 2B). The phenotype, either 'Koshihikari' or 'Kasalath', of 12 homozygous F_3 _plants derived from each F_2 _individual was determined. The chlorophyll contents in the first leaf below the flag leaf were used as an indicator of the lodging resistance during a typhoon; relative chlorophyll contents were determined as described in Ishimaru et al. [24]. The genotypes and phenotypes of F_3 _lines homozygote with respect to the *lrt5 *locus were investigated (Fig. 2C). Thus, we could narrow the *lrt5 *region down to 4.6 cM between markers L1008 and S0068 in one selection using 96 plants of an F_2 _population (Fig. 2).

### Application 2 (Multi-locus SNP genotyping)

As an example of multi-locus SNP genotyping, we made a set of 96 ASP-PCR markers (ASP array). We tested various ASP markers using 'Koshihikari' and 'Kasalath' genomic DNA, and the primers which ensure the allele specific amplification were collected and arrayed on two 96-well PCR plates according to chromosomal order. The 96 markers were separated on average by a distance of 3.9 Mbp (see Additional file 2: The list of ASP based on SNPs between 'Koshihikari' and 'Kasalath'.). The PCR condition was standardized among the ASP markers, and this enabled genome-wide SNP mapping in a single PCR operation.

#### Sub-protocol for multi-locus SNP genotyping

1. Prepare a PCR master mix by scaling the volumes listed below to the desired number of amplification reactions. Include 10% overage to cover pipetting errors.

• Add the following components to a nuclease-free microcentrifuge tube:

12.3 μl nuclease-free water

2 μl 10 × PCR buffer

1.6 μl 2.5 mM dNTP mix

1 μl sample DNA (ca. 200 ng)

0.1 μl Hot Goldstar DNA polymerase

• Mix gently and centrifuge to bring solution to the bottom of the tube.

2. Aliquot 19 μl of the PCR master mix into 96-well PCR plate and add 1 μl forward and reverse primer mix (10 μM).

**NOTE: ***The forward and reverse primers are mixed and the set of ASP primers are arranged in 96-well plate format beforehand*.

3. Place reactions in a thermal cycler heat block and incubate 10 min denaturation at 95°C; 30 cycles of 30 s denaturation at 95°C, 30 s annealing at 58°C, 45 s extension at 72°C; and 4 min final extension at 72°C.

4. Add 2 μl 10 × SYBR Green I to the PCR product, and acquire the fluorescence value using a real-time thermal cycler at 75°C.

5. Draw the graphical genotypes using Microsoft Excel macros based on genotype data and physical position of each SNP (to obtain these macro files, please contact to kenshi@nias.affrc.go.jp).

**Figure 3:**
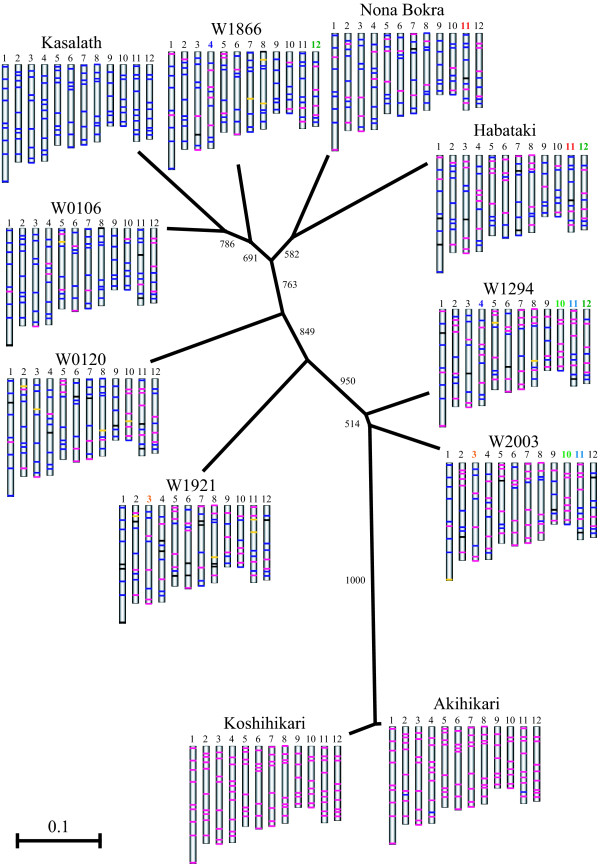
**An example of multi-locus SNP genotyping**. Graphical genotypes of 11 lines arranged in a neighbor-joining tree created on the basis of the 96-SNPs genotype. Numbers on branches represent bootstrap values with 1,000 replicates. The bars shown in red, blue and yellow on chromosomes represent homozygous 'Koshihikari' alleles, homozygous 'Kasalath' alleles and heterozygous alleles, respectively, while black bars indicate marker that could not be determined. Chromosome numbers shown in identical color in different plant lines indicate chromosomes sharing the same SNP pattern.

According to sub-protocol for multi-locus SNP genotyping, we tested ASP array in 11 rice and wild rice lines: two *japonica *cultivars ('Akihikari' and 'Koshihikari'), three *indica *cultivars ('Kasalath', 'Habataki' and 'Nona Bokra') provided by the Rice Genome Resource Center http://www.rgrc.dna.affrc.go.jp/index.html of the National Institute of Agrobiological Science (Tsukuba, Japan) and six accessions of *O. rufipogon *('W0106', 'W0120', 'W1294', 'W1866', 'W1921' and 'W2003') listed in the core collection of wild rice provided by the National Institute of Genetics (Shizuoka, Japan) supported by the National Bioresource Project, MEXT, Japan. The genome-wide graphical genotype maps of 11 lines were arranged in a neighbor-joining tree (Fig. 3; see also Additional file 3: Ninety-six SNPs genotypes of 11 plants analyzed by ASP array). The neighbor-joining tree was created with ClustalX [25] and NJplot [26] with bootstrap values > 500 (1,000 replicates). Among 96 SNPs in these rice cultivars and accessions, more than 90% of SNPs could be identified as either 'Koshihikari' or 'Kasalath' alleles, except for 'W1921'.

## Comments

### Effects of base mismatches on allele specificity

To improve the specificity of allele-specific amplification, single nucleotide artificial mismatches were introduced at the third nucleotide from the 3' end of the primers. The effects of base pair mismatches were tested using isolated genomic DNA and 10 different allele-specific primers for each artificial mismatch. After PCR amplification, the products were detected using EtBr after agarose gel electrophoresis, and the number of primers which shows single-band specific amplification to 'Koshihikari' or 'Kasalath' genomic DNA were counted (Table 1). At the third nucleotide from the 3' end matching bases (rows) were replaced in the primers by the bases shown in columns, primers without mismatches showed no allele specificity (underlined in Table 1). Hayashi et al. (2004) [17] proposed that base pair mismatches created through T-G or C-A transversions at third base from 3' end could increase the allele-specificity. We confirmed that these mismatched base pairs were useful for allele-specific amplification, and identified another possible mismatch base pair, A-T transversion and A-G transition. Thus, A-G transition as well as A-T, A-C, and G-T transversions were useful base pair mismatches to improve allele-specific amplification. Primer sequences (GC content) are determinant of melting temperature (Tm) which critical importance in designing the PCR condition. By introducing such artificial mismatches, Tm of allele-specific primers can be adjusted to standardized PCR conditions.

**Table 1:** Effect of artificial base mismatches on the specificity of allele-specific PCR.

		Mismatching base			
		**A**	**T**	**G**	**C**

**Matching base**	**A**	0%	50%	60%	60%
		(0/10)	(5/10)	(6/10)	(6/10)
	
	**T**	60%	0%	90%	10%
		(6/10)	(0/10)	(9/10)	(1/10)
	
	**G**	50%	90%	0%	40%
		(5/10)	(9/10)	(0/10)	(4/10)
	
	**C**	60%	40%	40%	0%
		(6/10)	(4/10)	(4/10)	(0/10)

### Flexibility of the ASP-PCR

ASP-PCR methods developed in this study is a simple method for multi-sample or multi-locus SNP genotyping. The method could be established easily in any molecular biology laboratory. New primer sets can be designed specifically to meet the requirements of various research purposes. For example, recombination events between two ASP markers could be easily detected (Fig. 2). The large-scale selection using ASP markers will greatly facilitate the mapping of genes or marker-assisted selection. On the other hand, multi-locus SNP mapping is enabled using sets of 96 ASP markers (Fig. 3). The multi-locus mapping will be useful in the high-resolution mapping after detection of various QTLs [27,28]. The ASP markers can be switched in and out by individual researchers, unlike a printed array. This flexibility is a major advantage of the ASP-PCR method.

### Applicability of ASP to diverse varieties

To demonstrate the applicability of ASP, we performed multi-locus genotyping in five cultivars of *O. sativa *and six accessions of *O. rufipogon*. *O. rufipogon *is the wild progenitor of *O. sativa*, *japonica *and *indica *cultivars are thought to have been domesticated from *O. rufipogon *independently [29]. Thus, the genetic variations of *O. sativa *might have their basis in the variations of *O. rufipogon*. We analyzed six accessions of *O. rufipogon *which are listed as the core collection (Rank 1) of wild rice [30], and two *O. sativa *ssp. *indica*, 'Habataki' and 'Nona Bokra', which are widely used as genetic resources for the genetic improvement of *japonica *rice. In these cultivars and accessions (except for 'W1921'), more than 90% of the alleles could be determined as either 'Koshihikari' or 'Kasalath' alleles through ASP array analysis (Fig. 3; see also Additional file 3). The average genotype call rate of 93.6% was achieved across 11 lines.

Intriguingly, in some *O. rufipogon *and *O. sativa*, some chromosomes shared identical SNP combinations, as indicated by the color-coded chromosome numbers in Fig. 3. For example, SNP genotypes on chromosome No. 12 was identical in 'Habataki', 'W1866' and 'W1294'. Due to the limited resolution of our markers, it is unclear whether the continuous chromosome regions are shared in some lines or not. Monna et al. (2006) [12] suggested that the genome of cultivated rice consists of a mosaic of various chromosomal segments derived from various accessions of wild rice. Further high-resolution mapping using the ASP array method in collections covering a wider range of genetic variation of *O. sativa*, as provided e.g. by the Rice Diversity Research Set of germplasm (RDRS) [31], may support the clarification of the domestication process of rice. The ASP array method will help to accelerate the genetic diversity analysis.

### Validity of SNP genotypes determined by ASP array analysis

The varieties of domesticated rice and wild rice might carry mutations in priming sites that could affect allele-specificity. To test for the presence of such unanticipated changes in priming sites and to validate the applicability of the ASP methodology, we sequenced the priming sites of unintentionally and randomly chosen 12 allele-specific PCR markers located at the distal end of 12 chromosomes in six accessions of *O. rufipogon *(see Additional file 4: The DNA sequences of upper priming site of 12 allele-specific PCR markers). We identified five unanticipated additional SNPs in a total of 72 sequences. Although some accessions have unanticipated SNPs at the upper priming site, there was 100% concordance between the SNP genotypes identified by ASP-PCR analysis and DNA sequence, except for two samples that could not be sequenced. These results indicated that ASP analysis enabled SNP determination in the presence of additional SNPs in priming sites.

### A comparison of existing methods and advantages of our method

Various methods for SNP analysis are available (reviewed in Shi [32]; Syvänen [33]), including TaqMan [34], oligonucleotide arrays [35], a fluorescence polarization method [36], pyrosequencing [37], and MassArray [38]. These methods enable high-throughput SNP analysis without electrophoresis. However, all these methods require significant initial investments for expensive probes, micro-chips, or special instrumentation. In Taqman array and oligonucleotide array, the markers cannot be replaced or added after establishment of the system. A gel-free SNP genotyping method with allele-specific PCR, BAMPER, has also been reported [39]. This method has flexibility and is cost-effective, it requires purified double strand PCR product and multi-step sample processing. SNP analysis generally has problems such as significant initial investments, inflexibility or labor-intensive.

In our ASP-PCR method, these problems are solved. Our ASP methodology allows gel-free SNP genotyping without expensive probes or specialized equipment. This method is highly versatile due to the simplicity and flexibility, and the method could be established easily in any molecular biology laboratory.

## Conclusion

We developed a simple and flexible method for SNP genotyping by improving the design of allele-specific PCR primers, establishing a method for the detection of PCR products without electrophoresis, and optimizing conditions for allele-specific PCR. We developed a total of 101 ASP markers, which could be applied to diverse cultivars. The novel ASP-PCR technique introduced here will greatly facilitate the SNP typing and gene mapping.

## Competing interests

The authors declare that they have no competing interests.

## Authors' contributions

NH and KI conceived the project, designed experiments, and prepared the manuscript, and NH carried out the experiments. NM carried out the acquisition and processing of fluorescence data, and helped the primer design. TK and KU participated in the DNA extraction and the phenotypic evaluation of *lrt5*. All authors contributed to the manuscript preparation, and approved its final version.

## Supplementary Material

Additional file 1 Allele-specific PCR primers used to map the *lrt5*. Click here for file

Additional file 2 The list of ASP based on SNPs between 'Koshihikari' and 'Kasalath'. Click here for file

Additional file 3 Ninety-six SNPs genotypes of 11 plants analyzed by ASP array. Click here for file

Additional file 4 The DNA sequences of upper priming site of 12 allele-specific PCR markers. Click here for file

## References

[B1] Konieczny K Ausubel FM A procedure for mapping *Arabidopsis *mutations using co-dominant ecotype-specific PCR-based markers Plant J 1993 4 403 410 10.1046/j.1365-313X.1993.04020403.x 8106085

[B2] Harushima Y Yano M Shomura A Sato M Shimano T Kuboki Y Yamamoto T Lin SY Antonio BA Parco A Kajiya H Huang N Yamamoto K Nagamura Y Kurata N Khush GS Sasaki T A high-density rice genetic linkage map with 2275 markers using a single F_2 _population Genetics 1988 148 479 494 10.1093/genetics/148.1.479 PMC1459786 9475757

[B3] McCouch SR Teytelman L Xu Y Lobos KB Clare K Walton M Fu B Maghirang R Li Z Xing Y Zhang Q Kono I Yano M Fjellstrom R DeClerck G Schneider D Cartinhour S Ware D Stein L Development and mapping of 2240 new SSR markers for rice (*Oryza sativa *L.) DNA Res 2002 9 199 207 10.1093/dnares/9.6.199 12597276

[B4] Shen YJ Jiang H Jin JP Zhang ZB Xi B He YY Wang G Wang C Qian L Li X Yu QB Liu HJ Chen DH Gao JH Huang H Shi TL Yang ZN Development of genome-wide DNA polymorphism database for map-based cloning of rice genes Plant Physiol 2004 135 1198 1205 10.1104/pp.103.038463 15266053 PMC519040

[B5] Jander G Norris S Rounsley S Bush D Levin I Last R *Arabidopsis *map-based cloning in the post-genome era Plant Physiol 2002 129 440 450 10.1104/pp.003533 12068090 PMC1540230

[B6] Zhu YL Song QJ Hyten DL Van Tassell CP Matukumalli LK Grimm DR Hyatt SM Fickus EW Young ND Cregan PB Single-nucleotide polymorphisms in soybean Genetics 2003 163 1123 1134 12663549 10.1093/genetics/163.3.1123 PMC1462490

[B7] Batley J Barker G O'Sullivan H Edwards KJ Edwards D Mining for single nucleotide polymorphisms and insertions/deletions in maize expressed sequence tag data Plant Physiol 2003 132 84 91 10.1104/pp.102.019422 12746514 PMC166954

[B8] International Rice Genome Sequencing Project The map-based sequence of the rice genome Nature 2005 436 793 800 10.1038/nature03895 16100779

[B9] Yu J Hu S Wang J Wong GK Li S Liu B Deng Y Dai L Zhou Y Zhang X Cao M Liu J Sun J Tang J Chen Y Huang X Lin W Ye C Tong W Cong L Geng J Han Y Li L Li W Hu G Huang X Li W Li J Liu Z Li L Liu J Qi Q Liu J Li L Li T Wang X Lu H Wu T Zhu M Ni P Han H Dong W Ren X Feng X Cui P Li X Wang H Xu X Zhai W Xu Z Zhang J He S Zhang J Xu J Zhang K Zheng X Dong J Zeng W Tao L Ye J Tan J Ren X Chen X He J Liu D Tian W Tian C Xia H Bao Q Li G Gao H Cao T Wang J Zhao W Li P Chen W Wang X Zhang Y Hu J Wang J Liu S Yang J Zhang G Xiong Y Li Z Mao L Zhou C Zhu Z Chen R Hao B Zheng W Chen S Guo W Li G Liu S Tao M Wang J Zhu L Yuan L Yang H A draft sequence of the rice genome (*Oryza sativa *L. ssp *indica*) Science 2002 296 79 92 10.1126/science.1068037 11935017

[B10] Nasu S Suzuki J Ohta R Hasegawa K Yui R Kitazawa N Monna L Minobe Y Search for and analysis of single nucleotide polymorphisms (SNPs) in rice (*Oryza sativa*, *Oryza rufipogon*) and establishment of SNP markers DNA Res 2002 9 163 171 10.1093/dnares/9.5.163 12465716

[B11] Feltus FA Wan J Schulze SR Estill JC Jiang N Paterson AH An SNP resource for rice genetics and breeding based on subspecies indica and japonica genome alignments Genome Res 2004 14 1812 1819 10.1101/gr.2479404 15342564 PMC515328

[B12] Monna L Ohta R Masuda H Koike A Minobe Y Genome-wide searching of single-nucleotide polymorphisms among eight distantly and closely related rice cultivars (*Oryza sativa *L.) and a wild accession (*Oryza rufipogon *Griff.) DNA Res 2006 13 43 51 10.1093/dnares/dsi030 16766512

[B13] Cho RJ Mindrinos M Richards DR Sapolsky RJ Anderson M Drenkard E Dewdney J Reuber TL Stammers M Federspiel N Theologis A Yang WH Hubbell E Au M Chung EY Lashkari D Lemieux B Dean C Lipshutz RJ Ausubel FM Davis RW Oefner PJ Genome-wide mapping with biallelic markers in Arabidopsis thaliana Nat Genet 1999 23 203 207 10.1038/13833 10508518

[B14] Newton CR Graham A Heptinstall LE Powell SJ Summers C Kalsheker N Smith JC Markham AF Analysis of any point mutation in DNA. The amplification refractory mutation system (ARMS) Nucleic Acids Res 1989 17 2503 2516 10.1093/nar/17.7.2503 2785681 PMC317639

[B15] Cha RS Zarbl H Keohavong P Thilly WG Mismatch amplification mutation assay (MAMA): amplification to the c-H-*ras *gene PCR Method Appl 1992 2 14 20 10.1101/gr.2.1.14 1490171

[B16] Kwok S Kellog DE McKinney N Spasic D Goda L Levenson C Sninsky JJ Effects of primer-template mismatches on the polymerase chain reaction: Human immunodeficiency virus type q model studies Nucleic Acids Res 1990 18 999 1005 10.1093/nar/18.4.999 2179874 PMC330356

[B17] Hayashi K Hashimoto N Daigen M Ashikawa I Development of PCR-based SNP markers for rice blast resistance genes at the *Piz *locus Theor Appl Genet 2004 108 1212 1220 10.1007/s00122-003-1553-0 14740086

[B18] Drenkard E Richter BG Rozen S Stutius LM Angell NA Mindrinos M Cho RJ Oefner PJ Davis RW Ausubel FM A simple procedure for the analysis of single nicleotide polymorphisms facilitates map-based cloning in Arabidopsis Plant Physiol 2000 124 1483 1492 10.1104/pp.124.4.1483 11115864 PMC1539302

[B19] Wei B Jing R Wang C Chen J Mao X Chang X Jia J *Dreb1 *genes in wheat (*Triticum aestivum *L.): development of functional markers and gene mapping based on SNPs Mol Breeding 2009 23 13 22 10.1007/s11032-008-9209-z

[B20] Shirasawa K Monna L Kishitani S Nishio T Single nucleotide polymorphisms in randomly selected genes among *japonica *Rice (*Oryza sativa *L.) varieties identified by PCR-RF-SSCP DNA Res 2004 11 275 283 10.1093/dnares/11.4.275 15500252

[B21] Murray MG Thompson WF Rapid isolation of high molecular weight plant DNA Nucleic Acids Res 1980 8 4321 4325 10.1093/nar/8.19.4321 7433111 PMC324241

[B22] Zipper H Brunner H Bernhagen J Vitzthum F Investigation on DNA intercalation and surface binding by SYBR Green I, its structure determination and methodological implications Nucleic Acids Res 2004 32 e103 10.1093/nar/gnh101 15249599 PMC484200

[B23] Vitzthum F Geiger G Bisswanger H Brunner H Bernhagen J A quantitative fluorescence-based microplate assay for the detection of double-strand DNA using SYBR Green I and a standard ultraviolet transilluminator gel imaging system Anal Biochem 1999 276 59 64 10.1006/abio.1999.4298 10585744

[B24] Ishimaru K Togawa E Ookawa T Kashiwagi T Madoka Y Hirotsu N New target for rice lodging resistance and its effect in a typhoon Planta 2008 227 601 609 10.1007/s00425-007-0642-8 17960419

[B25] Thompson JD Gibson TJ Plewniak F Jeanmougin F Higgins DG The ClustalX windows interface: flexible strategies for multiple sequence alignment aided by quality analysis tools Nucleic Acids Res 1997 24 4876 4882 10.1093/nar/25.24.4876 PMC147148 9396791

[B26] Perrière G Gouy M WWW-Query: An on-line retrieval system for biological sequence banks Biochimie 1996 78 364 369 10.1016/0300-9084(96)84768-7 8905155

[B27] Ishimaru K Yano M Aoki N Ono K Hirose T Lin SY Monna L Sasaki T Ohsugi R Toward the mapping of physiological and agronomic characters on a rice function map: QTL analysis and comparison between QTLs and expressed sequence tags Theor Appl Genet 2001 102 793 800 10.1007/s001220000467

[B28] Madoka Y Kashiwagi T Hirotsu N Ishimaru K Indian rice "Kasalath" contains genes that improve traits of Japanese premium rice "Koshihikari" Theor Appl Genet 2008 116 603 612 10.1007/s00122-007-0693-z 18097643

[B29] Londo JP Chiang YC Hung KH Chiang TY Shaal BA Phylogeography of asian wild rice, *Oryza rufipogon*, reveals multiple independent domestications of cultivated rice, *Oryza sativa* Proc Natl Acad Sci USA 2006 103 9578 9583 10.1073/pnas.0603152103 16766658 PMC1480449

[B30] Kurata N Yamazaki Y Oryzabase. An integrated biological and genome information database for rice Plant Physiol 2006 140 12 17 10.1104/pp.105.063008 16403737 PMC1326027

[B31] Kojima Y Ebana K Fukuoka S Nagamine T Kawase M Development of an RFLP-based rice diversity research set of germplasm Breed Sci 2005 55 431 440 10.1270/jsbbs.55.431

[B32] Shi MM Enabling large-scale pharmacogenetic studies by high-throughput mutation detection and genotyping technologies Cli Chem 2001 47 164 172 11159763

[B33] Syvänen AC Accessing genetic variation: Genotyping single nucleotide polymorphism Nat Rev Genet 2001 2 930 942 10.1038/35103535 11733746

[B34] Livak KJ Flood SJA Marmaro J Giusti W Deetz K Oligonucleotides with fluorescent dyes at opposite ends provide a quenched probe system useful for detecting PCR product and nucleic acid hybridization PCR Method Appl 1995 4 357 362 10.1101/gr.4.6.357 7580930

[B35] Hacia JG Sun B Hunt N Edgemon K Mosbrook D Robbins C Fodor SPA Tagle DA Collins FS Strategies for mutational analysis of the large multiexon *ATM *gene using high-density oligonucleotide arrays Genone Res 1998 8 1245 1258 10.1101/gr.8.12.1245 9872980

[B36] Chen X Levine L Kwok PY Fluorescence polarization in homogeneous nucleic acid analysis Genome Res 1999 9 492 498 10330129 PMC310763

[B37] Alderson A Kristofferson A Hammerling U Determination of single-nucleotide polymorphisms by real-time pyrophosphate DNA sequencing Genome Res 2000 10 1249 1258 10.1101/gr.10.8.1249 10958643 PMC310924

[B38] Jurinke C Boom D van den Cantor CR Koster H Automated genotyping using the DNA MassArray technology Method Mol Biol 2001 170 103 116 10.1385/1-59259-234-1:103 11357675

[B39] Zhou GH Kamahori M Okano K Chuan G Harada K Kambara H Quantitative detection of single nucleotide polymorphisms for a pooled sample by a bioluminometric assay coupled with modified primer extension reactions (BAMPER) Nucleic Acids Res 2001 29 e93 10.1093/nar/29.10.2003 11574695 PMC60253

